# Ten minutes to midnight: a narrative inquiry of people living with dying with advanced copd and their family members

**DOI:** 10.1080/17482631.2021.1893146

**Published:** 2021-03-08

**Authors:** Anita E. Molzahn, Laurene Sheilds, Marcy Antonio, Anne Bruce, Kara Schick-Makaroff, Robyn Wiebe

**Affiliations:** a Faculty of Nursing, University of Alberta, Edmonton, AB, Canada; b School of Nursing, Division of Teaching and Learning Support and Innovation, University of Victoria, Victoria, BC, Canada; c School of Nursing and School of Health Information Science, University of Victoria, Victoria, BC, Canada; d School of Nursing, University of Victoria, Victoria, BC, Canada

**Keywords:** Nursing, chronic obstructive pulmonary disease, family, chronic illness, narrative inquiry, qualitative research, end-of-life

## Abstract

**Purpose**: To explore how people with end stage chronic obstructive pulmonary disease and their family members describe living in the face of impending death.

**Methods**: A narrative inquiry was undertaken using a social constructionist perspective. Data were collected in 2017–18 in two in-depth interviews, lasting 90 to 120 minutes approximately 3–4 months apart, with a telephone follow-up 2–3 months later. Thematic analysis was conducted including analysis within and across participants.

**Results**: Sixteen people with advanced chronic obstructive pulmonary disease and seven family members participated. For both people with the disease and family members, six key themes/storylines emerged including missing life, being vigilant, hope and realism, avoiding death talk, the scary dying process, and need to prepare.

**Conclusion**: This study highlighted six key storylines about death and dying with advanced chronic obstructive pulmonary disease for people with the illness and their family members. The participants with the illness and their family members held similar perceptions about end of life. More supports are needed for people with advanced chronic obstructive pulmonary disease and their family members in living with their illness while ensuring that they experience a “good death.”

## Introduction

Chronic obstructive pulmonary disease (COPD) is the fourth leading cause of death globally (World Health Organization [WHO], 2020). Despite severe symptom burden in end stage COPD (Meharry & Anderson, 2016), in most regions and countries, people with this disease do not receive adequate access to palliative and end of life (EOL) care (Bloom et al., 2018; Braço Forte & Sousa, 2017; Lilly & Senderovich, 2016). Clearly, there are opportunities to enhance care of this population at this difficult time.

Most research on COPD has focused on aetiology and clinical treatments, and relatively few qualitative researchers have explored the perspectives of people living with advanced stages of COPD and family members helping to care for someone with advanced COPD (Cruz et al., 2017; Ek et al., 2015; Gatti et al., 2018; Graney et al., 2017; Malcolm et al., 2017; Marx et al., 2016; Strang et al., 2018). Qualitative research offers opportunities to better understand the perspectives of people with COPD and their family members. Hence, in this narrative inquiry, we explored living with advanced COPD in the face of impending death with this population. To our knowledge, this is the only study to explore experiences of both people with advanced COPD and their family members over time in a longitudinal study.

## The COPD experience

The symptom experience of people with COPD has been well described (Meharry & Anderson, 2016; Wu et al., 2018). Breathlessness or dyspnoea is a significant concern to those living with end-stage COPD (Miravitlles & Ribera, 2017). Ek et al. (2015) reported that some participants found the expectoration of sputum to be one of the most challenging and embarrassing of all symptoms. Glasser et al. (2016), in a focus group study, found that participants were frustrated that they could barely manage routine household activities such as making a bed and doing dishes, yet the disease was invisible to others. Despite availability of staging criteria, it is not always clear when someone with COPD is approaching EOL (Braço Forte & Sousa, 2017). Further, people with end stage COPD are far less likely to obtain palliative care than people with the same symptoms and illness profile with lung cancer (Butler et al., 2020; Kendzerska et al., 2019). Over the past decade, there has been increased recognition that health professionals need to develop better approaches for management of people suffering from advanced COPD. Yet, the slow onset of the symptoms and the “invisibility” of the disease and its symptoms, make it less likely that people with COPD will seek and obtain optimal health and EOL care.

In a narrative review of the impact of COPD (at various stages), family members reported negative effects of caring on their physical health, emotional, social, relational and financial/employment life dimensions (Cruz et al., 2017). They found few studies of how to support family caregivers (Cruz et al., 2017). Strang et al. (2019), in a qualitative study designed to describe perceptions of healthcare support relating to COPD from both the family caregivers’ (n = 36) and the staff’s (n = 17) perspective, found three themes: restricted everyday life, a changed relationship, and joy through adaption. Similarly, Ek et al. (2015) also reported caregiver stress and burden, but unexpectedly, all relatives described a peaceful death of their family member. During the final week of life, people with the illness either experienced a temporary improvement where death was unexpected or a continued deterioration where death was inevitable (Ek et al., 2015).

A deeper understanding of the experiences of people with COPD and their family caregivers can lead to better EOL care, which is especially important given the limited resources for non-cancer related EOL care (Lilly & Senderovich, 2016). In one population-based study in Ontario, Canada, people with COPD were found to be far less likely to receive palliative care than a sample of people with lung cancer (Kendzerska et al., 2019). The researchers found palliative care to be associated with fewer deaths in the acute care setting, fewer days in acute care, and less cost, regardless of whether the diagnosis was lung cancer or COPD (Kendzerska et al., 2019). Iyer et al. (2020), in a qualitative study of pulmonary and palliative care specialists, found that both groups of physicians agreed that early palliative care could have benefits for people with advanced COPD but that the pulmonary specialists had concerns about the risks of use of opioids and benzodiazepines. It seems that there are many barriers to palliative care in this population and strategies to support patients with severe COPD and their family members at EOL are needed.

## Research questions and approach

The research questions for the study were:

1. How do people diagnosed with advanced COPD describe living with impending death?
2. How do family members describe the experience of caring for a person diagnosed with advanced COPD and living with impending death?

### Design

This study was part of a larger study of the experiences of people with advanced chronic illness as they approached death (Molzahn et al., 2020, 2018). A narrative inquiry was undertaken using in-depth interviews to explore people’s experiences of living with the uncertainties of illness (specifically chronic kidney disease, cancer, heart failure, and COPD) and the likelihood of dying. In this paper, we focus on the sample of people with COPD and their family members. A social constructionist perspective framed the approach to the study. It is founded on the premise that peoples’ understandings and experiences of reality are sustained and shift through stories (Boyland, 2019; Burr, 2018). Narrative inquiry provides a lens into the multiple narratives and cultural/social discourses that shape how individuals experience illness.

## Methods

### Participants

People with advanced COPD and family members were purposively recruited because of the life-limiting state of their illness. A respiratory therapist from a home oxygen therapy programme identified people with advanced disease approaching EOL and invited them to participate in the study. The participants were told that the research team was interested in understanding the uncertainties of living with a serious illness. The inclusion criteria included: age over 18 years; and evidence of exacerbations, complications or progression of COPD (e.g., hospitalizations, greater inactivity, etc.). For the purposes of participation, spirometry was not used to verify the stage of illness. People were excluded if they had significant cognitive impairment or were not English-speaking. In the screening process, participants were asked to identify a family member who would be interested in participating. They were not excluded if they did not identify a person or if their family member chose not to participate.

### Ethical considerations

Ethical approval was granted by the Institutional Review Boards of the universities employing the researchers. Participation in the study was voluntary. Informed consent was obtained at the time of the first interview and was confirmed at each subsequent interview. Pseudonyms have been used to protect confidentiality of participants and care was taken to preserve confidentiality between members of the dyad. Given the sensitivity of the topic, a list of supports and resources was available to participants who needed additional emotional support.

### Data collection

Data for this study were collected in western Canada between September 2016 and September 2017. Three interviews, conducted by the same interviewer, were held. Four interviewers, including two doctoral students and two research assistants assisted with data collection. No prior relationships existed with any of the participants. All interviewers were female; two of the four interviewers were nurses and two held other health-related degrees. All were experienced interviewers. Narrative interviewing skills were further developed through training workshops and dialogue with the research team. Weekly debriefing meetings were held with interviewers and investigators, facilitating training, ensuring quality and familiarizing the team with the data.

The majority of first two in-depth interviews were conducted in person in the participant’s home at a mutually convenient time. One family member participated in the first and second interviews over the telephone. Interviews were approximately 90 to 120 minutes in length. The second interview enabled us to explore narratives and emerging storylines in greater depth after preliminary discussion and analysis by the research team. Findings did not change substantively between interviews, but greater depth of understanding was obtained. The third interview was held over the telephone and focused on clarifications and closure; the duration of the telephone interview was approximately 20 to 30 minutes. Whenever possible, participants in the dyad were interviewed individually to offer the opportunity for them to speak freely and confidentially. One family member had the participant with COPD present for the first in-person interview.

Interviews began with broad general questions such as, “Tell me about what it is like to live with a serious illness”, followed by questions guided by the specific comments and stories of the participants as well as the study objectives. Questioning for family members began with, “Tell me about what it’s like caring for your family member diagnosed with advanced COPD” and “How have your experiences of caring for your family member changed over time?” All interviews were audio-recorded and transcribed. Field notes were recorded after each interview and addressed nonverbal communication, context of the interview and notable changes in disease condition since the previous interview.

### Data analysis

Riessman’s (2008) approach to thematic analysis was used. Analysis included comparisons of perceptions of persons with COPD vis a vis their family members. We also looked for changes over time for each participant. By reading, re-reading and listening to the interviews, the research team came to know each participant and dyad. Two research assistants coded the data under the supervision of the research team. Any differences in coding or questions about categorization were discussed with the entire research team and after dialogue, decisions were made by consensus. Theoretical saturation was achieved when no new themes were identified and when participants’ descriptions became repetitive. N-Vivo^TM^ Version 12 software was used to facilitate coding of transcripts.

### Rigour

Trustworthiness was evaluated using the qualitative criteria of credibility, transferability, dependability and confirmability. Credibility was established through prolonged engagement with the participants who were purposively selected for the purposes of the study. Field notes were recorded after each interview. A detailed audit trail (Denzin & Lincoln, 2018) was maintained; records of all design and data analysis decisions were recorded, including the theoretical and process memos of analytic decisions. Memoing and discussion of issues of analysis within our team were means to reflexivity and critical to the integrity of this project. Member checking was not conducted. COREQ guidelines for reporting were used (Tong et al., 2007).

## Findings

### Participants

The 16 participants with advanced COPD ranged in age from 56 to 95 years of age (mean = 71.75) and all of them had one or more co-morbidities. These included: tuberculosis, atrial fibrillation, heart failure, cancer, depression, anxiety, HIV, Hepatitis C, aneurysm, fibromyalgia, arthritis, and agoraphobia. The seven family members ranged in age from 22 to 83 years (mean = 63.14 years). Eight of the 16 participants with COPD were male and eight were female. Four of the seven family members were female. They included five spouses (3 wives, 2 husbands) and two adult children. In terms of living arrangements, six participants with COPD lived with their partner/spouse, two lived with children, and eight lived alone (four of these in assisted living facilities).

One participant died after the first interview. Three participants withdrew from the study after the first interview; one couple withdrew because the person with illness was “too busy” with other things and another woman because she found it too difficult to talk about the illness. A daughter could not be reached after the first interview. In these instances, data from the first interview were included in the analysis.

### Themes/storylines emerging

A number of key themes/storylines relating to the uncertainties of living with dying were evident in the data. Participants shared powerful stories of living with COPD that related to the following themes: missing life, being vigilant, hope and realism, avoiding death talk, the scary dying process, and need to prepare.

### Missing life

All the participants with COPD told us stories of loss and regret about missing aspects of life. The disease clearly impeded their current ability to live a full life. Darren, a 56 year old man with emphysema commented, “my life is pretty empty here.” Similarly, Irene, a 76 year old woman, said, “I don’t miss … cigarettes. I just miss—miss my life.” The challenges of simply getting out of the house seemed insurmountable to Irene. “Because for me to take the oxygen with the cart, I would be tiring myself out immensely, trying to put the cart in the car, and then getting myself in.” Irene’s car represented her independence. She said, “There are days when it’s really bad, that I, you know. I just—I hope tonight, when I go to bed, I don’t wake up. And then when I do, I think, ‘Oh, well. Another day.’” Fred, a 95 year old man, talked about missing going out to restaurants and losing his independence. Shelley, a 57 year old woman with COPD, also talked about missing trips with her husband who was in a pipe band: “I’ve missed so many band trips … I am thinking he’s probably gonna take me in an urn and spread my ashes.” Similarly, Henry, an 85 year old man, talked about how he could no longer travel to a favourite vacation spot.

This sense of loss and regret extended to family members as well. Each of them spoke about what they missed by virtue of caregiving. April, a 76 year old spouse, talked about having to cancel a rail trip to visit with family and friends. She commented:

We miss travelling. We loved to go places and every so often, I forget and I said ‘why aren’t we going down to the warmer climate?’ Now that we have a few pennies … and then we realize … the insurance doesn’t cover his illness.

### Being vigilant

The participants with COPD vigilantly watched their symptoms for deterioration or signs of impending death. Carrie, a 60 year old woman who had frequent bouts of pneumonia said, “Well, I’m close to death, I guess. My lungs could shut down anytime. The last time I was in the hospital, my lungs were shutting down. But they pulled me through … And basically, it means that my lungs are really fragile.” She went on to say:

I get pneumonia all the time – I’m on antibiotics right now, … I started getting the yellow sputum with the blood in it. … We caught it early … . I was pretty depressed, ‘cause I thought it was gonna mean I’d be in the hospital again, ‘cause usually I do, with my pneumonia. Yeah. And they’ve done all the tests and everything, and my lungs look like Swiss cheese, they said. So I try to take life slowly now.

Rosie, aged 74, talked about looking at her eyes in the mirror, comparing them to the change she had noticed in her father’s and her partner’s eyes about a week before each of them died. She observed that her eyes seemed more watery than normal but otherwise fine, perhaps suggesting that it was not yet time to die. Sharon, a 66 year old woman, who also noticed a change in her eyes, said, “This is—this will be fatal to me, this disease in a very short while. You know, so … And it’s hard to get around. You’re very weak, you’re very tired. You lose your voice [laughing].” Most participants talked about their lack of energy and how they no longer felt strong. Darren said: “There are days I just … what am I getting out of bed for you know? And I kick myself out of bed. Put the air on. Slowly get out.”

The challenges of managing symptoms, oxygen therapy, and other treatments were mentioned by some participants. Further, the confounding symptoms of other co-morbid conditions, such as heart disease, made decision-making about going to the emergency department (ED) more difficult. Sharon said:

When I saw this respirologist … I explained to him that I didn’t know the difference between COPD and AFib anymore. [Chuckling] I said, ‘I need guidance.’ … And he told me that if I have AFib, I’m to take my own pulse – didn’t tell me what to listen for – to take my own pulse. And go to the Emergency … If it’s COPD, just stay home, because we can’t do anything for you, you’re on oxygen.

She went on to describe another incident where she had a fever, went to the ED, and was inappropriately given high levels of oxygen and an antibiotic to which she reacted. Other participants with COPD also struggled with the decision of when to seek care in the ED for COPD symptoms such as severe shortness of breath.

Family members also vigilantly observed their loved ones. They watched the type and nature of sleep, breathing, and mobility. Calvin, a 78 year old husband and caregiver, watched for cardiac symptoms in his wife. He was concerned because his wife’s father died from an embolism at a young age.

### Hope and realism

While all participants were realistic about death as the likely outcome of their disease, some participants held hope for a lung transplant. Shelley said, “the motivations are intermingled with, ‘I wonder about, if I do all this, maybe I will be a candidate and I will get to live longer.’” Nevertheless, these participants were realistic in that they reported they had seen other, sometimes younger healthier people, die after the surgery. Greta, a wife, talked about how her husband had improved: “So even with chronic diseases, you don’t give up hope.” When asked what her hope was for she sighed and tearily said, “an easy death.”

There was recognition among both participants with COPD and their family members that death was imminent. For instance, Carrie said, “I’m too young to die. I’m only 60 … Well, I’m close to death I guess. My lungs could shut down any time.” Elements of both hope and realism were evident in her conversations with us.

The realistic understanding about prognosis seemed, in most cases, to be the result of a COPD teaching program offered at the local hospital. Sharon offered unsolicited comments about this program:

It was the best thing I ever did. And they almost tell you what your life is gonna be like for the – and how it’s gonna end up. And they actually gave me an actuarial date, of when I will probably expire. And so far, they’re right on track.

She noted:

My biggest hurdle is going to be the fact that my purpose is not to extend my life as long as I possibly can. There’s no point in that when you have an illness like this. The illness wears you out.

Jake, a 58 year old man also realistically acknowledged his prognosis. He said, “I’ve got this illness that’s probably gonna kill me. I just don’t dwell on it”. He talked about coming to terms with his shortened life expectancy but noted, “I’m not to the point where they have to wipe my rear end cause I’d put a bullet in my head before I go to that point.” He noted that, “Physically, I’m not gonna get better. I’m gonna get worse.” Calvin, said “everybody ends, and it’s just like taxes, you know.” He and his wife used the metaphor of the Doomsday clock in relation to her impending death and described their state as “10 minutes to midnight”. He talked about how his wife’s doctor wanted her [wife] to sign a Do Not Resuscitate form but, “She’s not ready to do that obviously.”

### Avoiding death talk

Although the participants talked freely about death and dying, many of them told stories of others who couldn’t talk about their dying, particularly family members and healthcare providers. For instance, Rosie told us that when she said she could “pop off at any time,” her daughter said, “Don’t talk like that Mother.” This daughter avoided any conversations relating to her mother’s illness, whereas Rosie’s son made efforts to find information for her that wasn’t “scary.”

The participants in this study reported that the person most often reluctant to talk about dying was a health care provider. Ron, a 72 year old man who died during the course of the study, said:

But people don’t want to believe that, they don’t want to believe somebody’s dying. They don’t know how to respond … I remember the nurse sat right there [points to where interviewer is sitting]. And I said to her, “what is palliative care?” And she says, “You don’t want to know?” “Honey, be honest with me, that’s all I’m asking, is be honest with me.” She said, “they don’t live very long.” I said, “well, I’ve known for years that I wasn’t gonna live very long.”

Naomi, a 77 year old woman, said, “I said to him, ‘You know doctor, I think about dying’. He says ‘No, you don’t.’ I says, ‘yeah I–I do … ’ My doctor got upset with me.” Similarly, Darren’s doctor seemed to withdraw. Darren said, “Seems like he’s getting a little bit standoffish, almost … It just seems like he’s tired of me or something. I don’t know. When are you gonna die [Darren]?” [chuckles]. Facetiously, Darren said to his doctor, “I’ve never died before, you know.” Similarly, Sharon poignantly talked about how her respirologist looked out the window after handing her the advanced care planning guide.

### The scary dying process

Many of the participants said that they were not afraid of death itself but talked about the “scary” experiences of shortness of breath. Stan said:

That’s a scary experience, I’ll tell ya. You don’t wanna go through that. You know, because that – that’s not a fun thing. Well, it’s just like – you know, you’re – yeah – you know, you like – you like, they’re not quite sure whether you’re gonna die at that moment or not … . Cause you’re – you can’t breathe. Like your lungs are collapsed or something. It’s a very scary experience. You don’t wanna go through it. [Chuckling] And I’ve gone through that … mmm … couple of times in my life. You know, enough times to know that I DON’T LIKE IT. … I’m looking forward to – to ah … going to heaven and then meet Jesus – but I don’t want to suffer. You know what I mean? I don’t wanna suffer.

Many of our participants were concerned about how they might die with advanced COPD. Jake, a 58 year old man with COPD who had previously experienced pneumonia said:

I really don’t want to die by choking to death – that’s a nasty way to go [emphasis on nasty] … I don’t want to die like that. I really don’t, I don’t want to choke to death like being conscious and just choke to death …

Ron frequently referenced death during the interviews:

I do not look forward to dying. I sure do look forward to being dead though. I love the idea of me stopping breathing. Brain activity [clicks tongue]. Straight line. That moment, I will look forward to. But I know death can be a very ugly, very painful, very – unattractive. And I don’t want to bugger up the carpets here anymore than I have. Or the smell, you know, the smell goes, so I’ve told people, ‘you check on me every day’.

Jake had a number of “close calls” and had clearly thought about how he wished to die. Regarding pain control, he said:

To anesthetize myself to that degree, that’s not a good idea either … . Nod out and throw up in my sleep –That’s not a good way to go either … I don’t wanna end up like you know, in a vegetable situation, at all. I mean, I’ll take my own life at that point.

Rebecca, a 74 year old woman with asthma, did not want to be put on a ventilator. “I just wanna be left to go my own merry way. I think that most of us people that are short of breath and have problems breathing don’t … don’t want any heroics done.” Rosie said, “My parents lived in their house until they both died. I prefer it that way.”

Some participants talked about the possibility of medical assistance in dying which was legalized in Canada during the time frame of data collection for this study. Although we did not raise the topic, Darren noted, “They’ve got a new thing that came out, that—that—you know, right to die thing.” Sharon said, “I’m collecting all this stuff on the assisted dying. That’s something I’m seriously looking into.” She was reluctant to discuss the topic with her physician because she did not believe that he would be supportive. Carrie talked with her doctor about her preference for medical assistance in dying at home rather than in hospital. Irene talked about the process of removing her late husband from his respirator because there was no brain activity. She said that they had discussed such a situation and “neither of us EVER wanted to be like—a vegetable.” Others commented that they would not take this step for religious or other reasons, such as “It’s not my way of going.”

Family members also described the dying process as scary both for themselves and their family members. Greta stated, “He’s definitely afraid of what the end might be like.” She was afraid that he would give up because he was doing so poorly. When Rosie’s son was asked what was scary about the dying process, he said, “It sounds very final.” He went on to describe images that he had seen, perhaps in anti-smoking ads of someone “sort of in bed and … like hacking and connected to a machine and so forth” but didn’t want to think of his mother that way.

### Need to prepare

Both patient and family participants identified the need to prepare for death and its aftermath. Although Wade and Tara, a 67 year old man with COPD and his wife, had a number of general conversations about EOL, specific discussions about planning for a memorial service were difficult. Tara said:

Okay, well if anything happens to me, these are the songs that I, you know, that are important to me. And we’ve never even really talked about a funeral - … I was thinking more it would be like a wake where we would be having a beer and listening to vinyl records.

But when he came across the notebook where she listed his favourite songs,

He’s like, ‘What the hell is this?’ You know, he was totally twitched that I thought I’d kinda … already had one foot in the grave … that was an emotional time, because I was trying to explain to him that wasn’t the reason I did it. I did it because I was afraid of forgetting.

Tara also justified her planning by saying:

And Dr. ___ also said, ‘you know, you need to think about um, things, and make sure things are in place and talk to your family and decide whether or not this is the journey you wanna take’. And get your ducks in a row, basically.

On the other hand, Jake, a 58 year old man who had been a diver, put his will together and knew exactly how his remains were to be handled, “stuck in a piece of Plexiglas and dropped down to about 35 feet of water in—on a reef. Outside of territorial waters, and I want it done … . It’s a weird thing, but it’s so I’ll be diving joy.” Sharon talked about her anxiety about what would happen to her cat after death. During the second interview, she reported “contentment” that she had found a new home for her treasured pet in preparation for her own impending death. Rosie, when talking with her son about her possible death said, “Well you don’t need to worry. Everything’s in order. Everything’s in envelopes … won’t have any trouble finding anything.”

## Discussion

Participants with COPD and their family members openly and articulately talked about their lives and impending deaths. They described stories relating to missing life, being vigilant, hope and realism, avoiding death talk, the scary dying process, and need to prepare. Similar findings have been reported in other studies, but in less comprehensive ways.

Participants in this study told many stories of missing life. While the purpose of our study was not comparative, we were struck by the pervasiveness of isolation of participants with COPD in relation to other groups of people with advanced illness that we have interviewed (Molzahn et al., 2020, 2018). This isolation was initially evident during recruitment, when fewer than expected family members participated; many of the participants with COPD did not have a family member, or one who was willing to share their experiences. The isolation and challenges associated with missing life were also reported by others. Iyer et al. (2019) noted that 40% of people in an early palliative care program for COPD experienced social isolation. Marx et al. (2016) suggested volunteer support to address the isolation associated with the illness. This isolation may further be compounded by the lack of centralized health services for COPD as compared with other serious illnesses such as cancer and chronic kidney disease.

Given the participants’ vigilance over their illness, additional education regarding the illness trajectory and symptom management is an area for nurses to consider in care planning. Symptom management could be supported by a palliative care team (Maddocks et al., 2017). Despite their vigilance and the “scary” nature of symptoms and illness, participants reported that health professionals and family members seemed reluctant to discuss their fears, especially relating to EOL.

While it is important that health care providers provide realistic messages while maintaining hope of patients and family members, it seems that health care providers are often not comfortable discussing death and dying. Ek et al. (2015) also found that there was little support for psychosocial and existential needs of patients with COPD and their family members, but all family members reported that their deceased relatives had a peaceful death. This could be a reassuring message for people with COPD. Further pre- and post-licensure education regarding discussion of death and dying may, over time, help nurses and other health professionals talk more openly and meaningfully to people with advanced illness. There is no simple checklist on how to have these discussions. A relational approach is warranted (Keeley & Hunter, 2017).

Participants in this study talked about their need to prepare for EOL. In western Canada, like many other regions (Bloom et al., 2018; Ek et al., 2015; Lilly & Senderovich, 2016), supportive and/or palliative care programmes for people with COPD need further development and evaluation. While the potential of palliative care for non-cancer healthcare delivery has been recognized (Siouta et al., 2016), people living with COPD often do not perceive themselves as dying, and therefore do not see the need for palliative care. The uncertain nature of the disease makes it difficult for patients, caregivers, and healthcare professionals to recognize a distinct state of EOL and hesitant to discuss EOL care (Lilly & Senderovich, 2016; Siouta et al., 2016). Lilly and Senderovich (2016) argue that EOL care should be integrated with symptom management and overall disease-driven care. Integrating EOL care discussions and decisions with overall treatment plans would help to not only alleviate concerns about symptom management and the dying process (Lilly & Senderovich, 2016), but also create a more supportive experience for patients and caregivers. This can be done by employing a palliative approach (Reimer‐Kirkham et al., 2016; Sawatzky et al., 2016) through use of palliative care principles early in care planning to meet the needs of people with life‐limiting chronic conditions.

In this study, family members described similar experiences to participants with COPD and both family members and participants with illness reported limited informal and formal supports. Similarly, Strang et al. (2019) argued for family-centred care in relation to advanced COPD. In their qualitative study of health professionals and informal caregivers, Strang et al. (2019) found that informal caregivers needed emotional, practical and informational support but described unclear expectations and ambiguity that impeded the provision of support. Both caregivers and staff described positive experiences of dialogue and indicated that knowledgeable and perceptive communication is key to support.

There are several potential limitations to the study. Our sample is limited given that participants were referred by a single health professional. The findings may be specific to the context and might therefore not be transferable to other countries, or other healthcare systems. Further, care of people with COPD may have changed over the last few years since data were collected. Nine participants did not identify a family member to participate, so we were unable to compare fully across families.

Further research relating to the impact of EOL care in COPD on family caregivers is warranted. It is especially important to consider caregivers when developing and implementing of EOL programs for this population (Lilly & Senderovich, 2016). It seemed that these participants had limited social support and this could be explored in future studies.

## Conclusion

The findings from this study highlighted perspectives of participants with advanced COPD and their family members over time regarding living with dying with this serious illness. Key storylines that emerged from the data included missing life, being vigilant, hope and realism, avoiding death talk, the scary dying process, and need to prepare. Similar perspectives were noted for participants with illness and their family members.

Although findings from this study cannot be generalized and some observations may be unique to western Canada, it seems that there are a number of areas where nurses or other health care professionals could enhance care of people with COPD who are approaching EOL. Given concerns about shortness of breath and pain at EOL, teaching programs could better address what to expect in relation to symptoms and when to seek treatment and/or emergency care. Education of both health professionals and family members relating to EOL communication would also be valuable. In summary, while the symptom experience of people with advanced COPD is known and understood, person-centred care calls for a greater understanding of the perceptions of people with advanced COPD and their family members regarding life in the face of impending death. Their perspectives could inform development and enhancement of EOL care for this challenging journey.

## Data Availability

Due to the nature of this research, we do not have ethics committee approval to share the raw data of participants of this study, so supporting data is not available.
